# COVID-19 and its impact on the profit of mango value chain actors

**DOI:** 10.1371/journal.pone.0299572

**Published:** 2024-04-03

**Authors:** Edward Ebo Onumah, Bright Ketadzo, Abigail Ampomah Adaku, Justina Adwoa Onumah, Prince Addey Owusu

**Affiliations:** 1 Department of Agricultural Economics and Agribusiness, School of Agriculture, University of Ghana, Legon, Accra, Ghana; 2 Science and Technology Policy Research Institute, The Council for Scientific and Industrial Research, Cantonments, Ghana; Shanghai Ocean University, CHINA

## Abstract

The unprecedented impact of the pandemic on both activities and profit of actors draws out the various areas of the value chain that need to be strengthened to ensure resilience in the face of global shock. This study fills the gap by assessing the extent at which COVID-19 impacted the profit of mango value chain actors in southern Ghana. It also analyzed the governance structure and the existing linkages in the dissemination of market information in relation to the profit of the actors. A two-year panel survey on 240 respondents was conducted in 2020 through a multi-stage sampling technique in Greater Accra, Eastern and Volta regions of Ghana. Net Farm Income, Social Network Analysis and Difference-in-Difference models were used in analyzing the data. Findings revealed that mango processors have more bargaining power and make the most profit while producers receive more information than other actors. Farmer-based organizations were found to be the prominent node and influential in the dissemination of market information within the value chain. The outbreak of COVID-19 negatively impacted the profit of mango producers and distributors; however, processors had a positive impact on their profit. The study therefore demonstrated that producers and distributors were vulnerable to the effect of the COVID-19 shock, whilst processors were robust to the shocks. Thus, reformed policies by all stakeholders for emergency preparedness should be targeted especially at those vulnerable actors in the chain. Additionally, FBOs, retailers and other key stakeholders should be considered in policy development to enhance market information dissemination.

## Introduction

The fruit industry is a crucial sub-sector of the Ghanaian agricultural sector which has boosted food security and income generation for value chain actors. Despite its substantial contribution to the country’s Gross Domestic Product (GDP) [1], the sector is sensitive to internal shocks such as inadequate rainfall, unstable market prices, labour, and government policies among others. The coronavirus (COVID-19) as an external shock with a potentially varied impact on the horticultural value chain is of no means to be underestimated [2]. The COVID-19 was first identified in Wuhan, China in December 2019 [3] but Ghana recorded its first case in the second week of March, 2020 [4]. In March 2020, the World Health Organization (WHO) described the virus as a global pandemic [5] and hence needed a rigorous approach to contain its spread. The fast spread of the virus caused a lot of countries to collaborate with the WHO to put stringent measures in place to halt its impact and to minimize its implications on human, food value chain and society at large [6]. Lockdowns and travel restrictions both within and outside were key measures adopted which affected a lot of countries around the world, as penned by [7, 8].

The measures adopted by Ghana included travel restrictions, ban on public or social gatherings (funerals, weddings, and parties), closure of schools and churches, movie theatres, and gyms, closure of restaurants, pubs and shops, and restrictions on the movement of cargo trucks. These measures affected a lot of businesses in Ghana including agriculture from production fields to consumers [9, 10]. The situation affected the profits of value chain actors due to a reduction in production volumes, high input and transportation costs, and unreliable market among others. This invariably triggered an impact on the livelihood of people who relied on agricultural related activities for survival, generating a lot of interest among researchers [11] who predicted the pandemic as a significant threat and menace to the global economy, while others [3] delineated the pandemic as a means for emerging paradigm of food system activities.

According to [12], pandemics such as the COVID-19 presented unusual type of risk, which could render a value chain inefficient or inoperable for an uncertain time duration. It is asserted that the effect of the pandemic may stifle the value chain activities, especially from the production point to distribution hence, making the delivery and accessibility of quality products to consumers difficult. This could have devastated impact on all value chain actors including producers, processors, distributors, and consumers [13, 14]. To ensure availability and accessibility of production inputs and food for consumers, many actors resorted to increased price of items to enhance their profits [3]. However, in Kenya for instance, [15] identified that the outbreak of the COVID-19 had negative impact on household income (profit) of fruits and vegetable value chain actors, compelling some to diversify into other business activities to adapt to the shock.

Equally, [16] mentioned that the people in rural areas were severely affected by the pandemic in terms of their income and bargaining power relative to people in urban areas. This reflects the high vulnerability of rural livelihoods to economic shocks and over-dependence on the urban market. Producers, distributors, processors, and consumers who were put in an awkward state by the ripple effect of the pandemic had no choice but to adhere and adapt to the restrictions and regulations to navigate through the situation. The COVID-19 restrictions further limited the flow of information which is necessary for improvement in the profit of value chain actors. An economic slump in the value chain actors’ revenue which is a function of their profit due to the impact of COVID-19 is one interesting finding revealed by [5, 11]. In the same way, [17] pointed that the agricultural food supplier vulnerability to economic shocks due the COVID-19 prohibitions and closure of market facilities could ditch the profit of value chain actors. Hence, factors such as bargaining power and information asymmetry could worsen the disruption of the food supply as a result of the COVID-19 [18]. [19] noted that establishing a strong guarantee for minimizing the effects of pandemics, and ensuring strong collaboration among relevant industry stakeholders based on information integration and dissemination could offer precautionary measures against other future uncertainties.

The foregoing limitations posed by the COVID-19 and its implications on the operation of the mango value chain raise a concern for the analysis of COVID-19 and its impact on the profit of mango value chain actors in Ghana. These factors, together with the deficiency of some empirical studies on the performance of value chain actors in the mango industry underscore the timeliness and the need for this study. Though, some studies have looked at the impact of the COVID-19 on labour availability, business performance and the Ghanaian economy [5, 14, 20], the goal of this paper is to fill the knowledge gap by assessing the impact of COVID-19 on the profit and the governance structure of the mango value chain by providing empirical evidence that gives a better comprehension of how the actors could be resilient in the face of global shock.

## Materials and methods

This section presents the conceptual framework guiding the study, method of data analysis, data collection technique, variable, and survey area description.

### Conceptual framework

**Fig 1:**
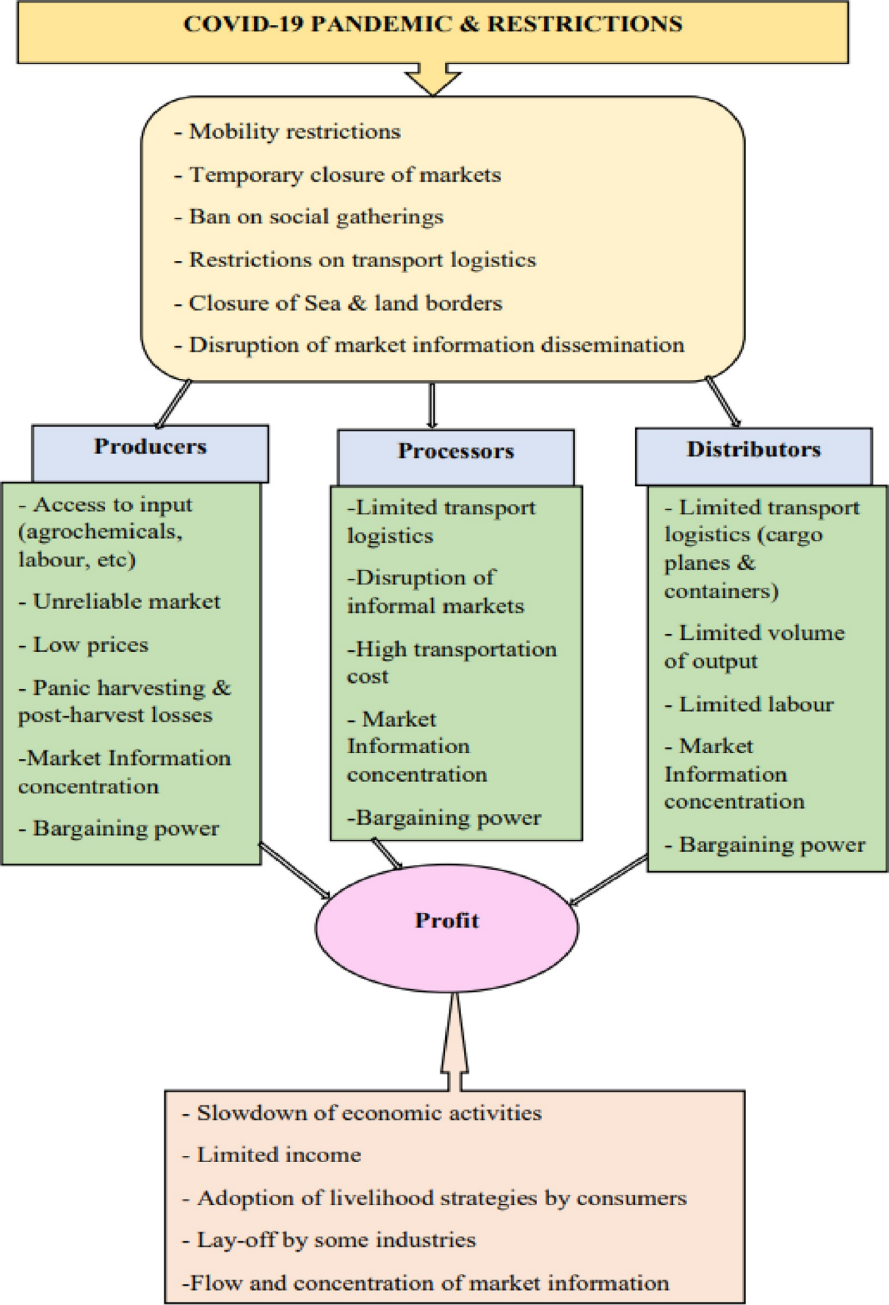
Conceptual framework, adapted from [21, 22].

The conceptual framework as shown in Fig 1 is adapted from [21, 22] with relevant information synthesized from [11, 14]. It reflects the impact of COVID-19 on the various sections of the value chain and its actors, which invariably affects their profit earned. It was captured by [5, 20, 23] that COVID-19 influenced and disrupted the activities of the value chain actors. From the producers’ side these include the disruption of input accessibility, unreliable market, low or uncertain prices for farm produce and panic-harvesting to avert heavy losses. The distributors experienced transport and logistics challenges, disturbance of informal markets and high transportation cost among others. Processors were also heavily affected by the limited number of cargo planes and containers at the air- and seaports respectively and limited labour which jointly reduced the volume of processed mango for sale in the domestic and export market. Accessibility and dissemination of market information during the outbreak of the COVID-19 are important factors that could strengthen the bargaining power of actors and hence, influence the profit generated. [21] argued that the implications of COVID-19 affected market information dissemination and slowed down economic activities which made some workers laid off.

### Survey area description

The survey was conducted in the Greater Accra, Eastern and Volta Regions of Ghana. Specifically, Yilo Krobo Municipal, Upper Manya Krobo Municipal and Okere District of the Eastern region, Shai Osudoku of the Greater Accra region, North Tongu Municipal, Ketu South Municipal and Kpando Municipal of the Volta region were the various areas selected and visited.

These areas were considered for the study due to the dominance of mango production, distribution, and processing in Ghana. Farming, especially mango production, is the primary occupation for most people living in the survey areas listed. Mango processors in these areas include both the commercial (for example, Blue Skies Company Ltd., Bomart, Eden Fruits, & HPW Fruits and Dry) and small-scale processors. The Greater Accra region, which was severely hit by COVID-19 and attracted three weeks of lockdown serves as the major market destination of those in the hinterlands (Volta & Eastern region).

#### Data collection

The paper employed mixed methods in the design of the research. The power analysis was considered to select the sample size from the three regions. Data was collected in May-June 2020 from 240 mango value chain actors (producers, distributors, and processors) with the aid of a structured questionnaire in a randomized manner. Respondents referred to their written records as much as possible to provide information on production, distribution and processing information including operating cost and revenue earned in 2019 and 2020, indicating before and during the outbreak of COVID-19. Some respondents (though very few) who did not have up-to-date records were asked to compliment information assessed through recall. Enumerators were well trained to ensure very accurate and efficient data collection. Also, focus group discussions and on-phone follow up interviews in some cases were conducted to validate information collected. COVID-19 protocols were adhered to as much as possible during the data collection. STATA and UCINET software were used in analyzing the data.

### Ethic statement

Prior to the data collection, ethical clearance was obtained from the seminar committee of the Department of Agricultural Economics, University of Ghana after thesis proposal presentation. Approval was obtained from all communities visited before the field data collection. All issues concerning the informed consent of study participants, anonymity and confidentiality were given serious consideration. Respondents were contacted individually, and verbal informed consent was obtained from all participants who were included in the research. Well trained enumerators comprehensively explained the objectives of the research, confidentiality procedures, risk and benefit involved, and the need to opt out of the survey at any time. An individual who decided to opt out of the research was excluded and never forced in any way to participate in the field exercises. All respondents were assured that their responses would be recognized or identified with unique codes.

### Method of analysis

The governance structure and the linkages that exist in the dissemination of market information within the mango value chain were analyzed using the scoring exercise and the social network analysis, respectively. The study also adopted the difference-in-difference model to examine the impact of the COVID-19 pandemic on the profit of mango value chain actors:

### The scoring exercise

Perception of actors on power relation in the mango value chain (governance structure) was investigated using the scoring exercise by [24] on selected variables (Profit, Information concentration and Bargaining power) which could make an actor exercise “importance” and “influence”. [22] revealed that “importance” is analyzed as an actor’s ability to exercise great effect and coordination within the chain, while “influence” is the power an actor possesses in exerting control and effect on other actors present in the chain. To assess this relationship, producers, distributors, and processors within the mango value chain were asked to score their position of ‘importance’ and ‘influence’ in relation to the selected variables. The higher the score (between zero and 100%), the higher the level of importance and influence, indicating dominance along the value chain. The score reflects the strength of the variable for each actor. The social network analysis was used to quantify the relations in the governance structure of the mango value chain in addition to the scoring exercise. This implies that the higher the score (%), the higher the level of importance and influence the actor carries hence, the dominance of that actor within the chain. This further suggests that an actor with the highest score for information concentration could have more strength to bargain for high prices or other incentives to maximize their profit.

### Social Network Analysis (SNA)

Social network analysis is an analytical tool that aids in measuring, visualizing, and simulating relationships or interactions among people or organizations as well as making mathematical analyses of these connectedness [25]. SNA is made up of a theoretical perspective which describes how the interactions of individual autonomous actors create the social structures of the community (say, value chain) whilst the analytical perspective assesses those interactions and social structures as networks of nodes (stakeholders) and ties (connections) [26]. The analytical aspects of SNA reveal the structure of social links and interactions, as well as the impact of those structures on other social phenomena to better understand the interaction among people, organizations, or other units of analysis [27, 28].

For this study, stakeholders that exist within the mango value chain and are involved in dissemination of market information were identified from literature and through focus group discussions. These stakeholders were asked questions that relate to the role(s), mode of operation, and connectedness in ensuring efficient distribution of current market information within the mango value chain. The data collected were compiled in a square (*nxn*) matrix form such that if there is an information sharing between stakeholder *m* and stakeholder *j*, the matrix element is assigned a value of 1; *n*_*mj*_ *= 1* or *n*_*jm*_ *= 1*. However, if there is no connection in terms of information sharing, it is given a value of 0; hence, *n*_*mj*_ *= 0* or *n*_*jm*_ *= 0*. However, the relationship is assumed not symmetric, as *n*_*mj*_ *= 1* does not imply *n*_*jm*_ *= 1*. Each respondent was asked to name any other stakeholder in the network that may share important information leading to market information dissemination. This technique allows a stakeholder, say a retailer to name an unlimited number of network members which help to distinguish between strong and weak relationships and simplifies the network for better acquisition of a detailed image of each network connections [29, 30]. [29] describes this approach as the socio-metric method to measure network links and interactions which was established by [30] and has been used by several network analysts including [31, 32].

Additionally, centrality indicators which include network density, betweenness centrality, and degree of centrality, were adopted to assess the network structure. Network density (D) is a proportion of all possible connections in a network. It calculates the number of stakeholders that are related to other stakeholders in the network while degree centrality according to [33] is the number of direct connections (ties) that a stakeholder (node) has with others, and hence it is a measure of activity. When the score of each of these measures is higher, it reflects how connected the stakeholder is in the network. This shows how influential or prominent they are in disseminating market information. The in-degree measure reflects how many interactions a stakeholder receives from other stakeholders thus, revealing their prominence in the network whilst the out-degree, on the other hand, assesses how well stakeholders can be joined to other network members hence, describing how influential they are [25]. Betweenness centrality, as defined by [34], is the number of times a stakeholder (node) functions as a bridge along the shortest path between two other network members or players. Eqs 1–4 reveal the centrality measures defined by [35] and adopted by [36].

$$Network\;density\;(D),\;D=\frac{\lambda }{\frac{N(N-\lambda )}{2}}$$ (1)

$$The\;degree\;centrality≔C_{d}(n_{i})=\lambda_{i}(n_{i})/(N‐1)$$ (2)

where *n*_*i*_ denotes the *i*^*th*^ actor(node) in the network, λ_*i*_*(n*_*i*_*)* denotes the number of connections (ties) to *n*_*i*_, and *N—1* represents the size of the network less the actor of interest.

Betweennesscentrality:CB(i)=∑h<kfhm(i)fhm (3)

$$normalized\;as:\;C_{B}(i)=C_{B}(i)/[\frac{\left(n-1)(n-2\right)}{2}]$$ (4)

where,

*C*_*B*_*(i)* = the betweenness centrality of the individual stakeholder

*g*_*jk*_ = the number of shortest path (geodesics connection) *jk*

*g*_*jk*_*(i)* = the number of geodesics that stakeholder *i* is on

*[(n-1)(n-2)/2]* = number of pairs of vertices (nodes) including the vertex itself

### Difference-in-difference model

The Difference in Difference (DID) model is an impact assessment tool that aids in the evaluation of public intervention and or other treatments of interest on some relevant outcome indicators [36]. The DID is measured as the difference between the average outcome changes over time in the intervention areas and the comparison areas [37]. The fundamental assumption of the DID is that subject to the other factors in the model, the changes over time in the intervention areas would have been identical to those in the comparison areas in the absence of the intervention. For the focus of this study, the DID estimates and associated standard errors are determined using the Ordinary Least Squares (OLS) in the panel survey from mango value chain actors in 2019 and 2020 (before and during COVID-19, respectively). The analysis evaluated the changes in profit earned in 2019 and 2020. The DID model for a standard member of any of the periods (either 2019 or 2020) is written as:

$$Z_{jkt}=\alpha_{0}+\alpha_{1}\lambda 2+a_{2}dQ+a_{3}\lambda 2*dQ+x_{t}+e$$ (5)

where *Z*_*jkt*_ represents the variable of interest (Profit) of respondent *j* in district *k* at time *t*, *dQ* is a dummy variable for the treatment status taking a value of one for invents in 2020 during the COVID-19 restrictions and zero for 2019, pre-COVID-19 period. Before the outbreak of the COVID-19 virus, the dummy variable *λ2* represents possible disparities between the treatment and control groups. Even in the absence of the change or the COVID-19 restrictions and shocks, the time-period dummy, *λ2*, represents aggregate elements that might induce changes in *Z*_*jkt*_. The coefficient of the interaction term λ2*dQ gives the DID estimates thus, revealing the effect of the COVID-19 restrictions and its shocks on the variable of interest, profit. A time-fixed effect term, x_t_ was introduced in the model to specify the time-specific effect across the treatment and control districts and *e* is the error term. For observations in the treatment group in the second period, this represents a dummy variable equal to one. The estimate for difference-in-differences is *a*_*3*_ [9, 38].

The difference-in-differences estimator could be calculated by taking the difference in one outcome before (t = 0) and during (t = 1) the outbreak of the COVID-19 disease and imposition of the restrictions e.g. *E(ΔY*_*Treat*_*) = E(Y*_*Treat1*_*) − R(Y*_*Treat0*_*)*.

Also, unrelated trends or the impacts related to COVID-19 shocks and restrictions was estimated by the difference in the outcome variable within the control (2019) and the treated (2020) periods as represented mathematically below:

$$Z(DID)=[Z(Y_{Treat1})-Z(Y_{Treat0})]–[Z(Y_{Control1})-Z(Y_{Control0})]=Z(\Delta Y_{Treat})-Z(\Delta Y_{Control})$$ (6)

The profit (the outcome variable in the DID model) of the producers, distributors and processors is examined by subtracting the total cost of production from the total revenue generated in 2019 and 2020, whilst using the Net Farm Income. This is illustrated mathematically in model 7:

Profit(NFI)=TotalRevenue(TR)−TotalCost(TC) (7)

Where Total Cost = Total Fixed Cost (TFC) + Total Variable Cost (TVC)

Variable cost elements include the cost of fertilizer and pesticides, labour, transportation, loading and unloading, market toll and other intermediary costs such as rented cost of land.

Fixed cost elements include depreciated value of (cutlass, spraying machine, pruning saw, pickaxe, crates, boxes, selling stands or tables, processing machine).

The fixed cost elements were depreciated using the straight-line depreciation method (8). The straight-line depreciation method was employed to determine the economic loss in the market value of the capital assets. Economic loss can be attributed to obsolescence and wear or tear.


$$Depreciation=\frac{Original\;cost–Salvage\;value}{Expected\;useful\;life}$$ (8)

The profit of actors was log-transformed to ensure linearity between the dependent and independent variable hence, improving the validity of the result. Similarly, [39] explained that although the magnitude of the variables from the first stage cannot be easily determined since the probit model is strictly monotonic in function, the interpretation of the signs of the interactive variables is simplified by the linearity of the dummies of the DID in the second stage. The margins command, which uses the fixed component of the model to estimate the magnitude of the estimated effect in terms of percentage points was used in this study to estimate the marginal effects.

### Variable description and justification for inclusion

The variable description and justification for inclusion are specified in Table 1. These include the following.

**Table 1:** Explanatory variables included in the difference-in-difference model.

Independent Variables	Description & Measurement	Variable Type	Expected sign		
			Producer	Distributor	Processor
Age	Years	Continuous	+	+	+
Gender	Dummy (1 = Male, 0 = Female)				
		Categorical			
Educational background	Number of years	Continuous	+	+	+
High cost of inputs	Dummy (Yes = 1, No = 0)	Categorical	-	+	+
Unit price in per tons	GHS	Continuous	+	+	+
Higher Output price	Dummy (Yes = 1, No = 0)	Categorical	+	+	+
Reliable customer	Dummy (Yes = 1, No = 0)	Categorical	+	+	+
Number of buyers	Dummy (Yes = 1, No = 0)	Categorical	+	+	+
Own transport	Dummy (Yes = 1, No = 0)	Categorical	+	+	+
Volume or quantity bought	Quantity in tonnes	Continuous		+	+
Farm size	Hectares	Continuous	+		
Cooperative/FBO Membership	(1 if Actor is a member of a cooperative; 0 otherwise)	Categorical	+	+	+
Value addition	(1 if Actor adds value to the mango; 0 otherwise)	Categorical		+	+

Source: Author’s construct

#### Age

It is a continuous variable and has been measured in years. The age of the respondents is expected to have a positive correlation with the choice of a specific marketing outlet by the value chain actors considered for this study. For example, it is expected that as age increases mango producers will tend to sell to a specific market outlet that maximizes their utility [40]. [41] discovered that age is positively connected with the decision to sell or not sell to a given marketing channel among cattle farmers in North-Central Namibia. Their study pointed out that older value chain actors like cattle producers might have transacted business with many of the marketing outlets and may know which outlet is more beneficial to earn more profit than the others.

#### Gender

The gender of the respondents is a dummy variable and is expected to positively influence the choice of a market outlet by the actors along the mango value chain (Producers, Distributors, and Processors). Male mango producers as revealed by [42], have better access to marketing information and productive resources necessary to meet the various quality requirements for a sustainable marketing channel than female farmers. For instance, most male mango producers have the largest farms and belong to farm cooperatives where relevant information are shared and have access to outlets that could increase their profit [42]. Hence, gender could possibly influence the decision of the mango value chain actors in their choice of marketing outlet.

#### High cost of inputs

High cost of inputs was measured as a dummy variable. It is expected that due to the higher cost of input value chain actors like producers, for example, will switch to sell to a market outlet that will maximize their profit to cover up for the cost involved in production. Since the mango value chain is both capital and labour intensive to ensure its effective running and comes with so much cost thus, it will be very necessary for many of the actors in the chain to produce while minimizing their cost.

#### Higher output price

Higher output price was measured as a dummy variable. [40] asserted that higher output price provides an incentive to the selling point. It is therefore expected that when the price of the mango fruit/product increases, the actors (producers, distributors, and processors) will tend to sell to outlets that maximize utility. For example, producers will choose to sell to processors since they offer a higher price and buy in large volumes. Smallholder farmers in Ghana selected market outlets based on product price, according to [39]. They established that whether a rural or an urban market was chosen may be controlled by the price of outputs. Higher output price serves as a great incentive for many actors like producers, distributors and processors. Hence, it is expected to influence profit positively.

#### Number of buyers

It was expected that many buyers of the mango product will positively influence the profit of the mango producers, distributors, and processors. As such, the number of buyers was a dummy variable. It was expected that the number of buyer concentration will affect the profit received by the value chain actors [42] during the outbreak of COVID-19.

#### Reliable customers

Reliable customers were measured as a dummy variable. Respondents who chose yes for agreeing that reliable customer influences their profit were assigned one while those who choose No were assigned zero. It was expected that regular customer relations will positively influence the choice of marketing outlet and profit of the mango producers, distributors, and processors.

#### Ownership of transport assets

Ownership of transport was expected to influence the choice of the local market, processors, and. This is because mango producers who own transport facilities could supply their products to local market centers and sell to retailers and even directly to consumers by obtaining better prices [43]. According to [44], having on-farm transportation enhances the likelihood of transferring goods to private merchants and retailers in the market.

#### Education

Education was measured as a dummy variable to avoid a degree of freedom trap. It is expected that being educated will positively influence the choice of the value chain actors considered. This is because these actors can gather and understand production and marketing information so that they can adjust their production and marketing systems according to the different market demands, hence, can be good negotiators. Farmers who are educated, for example, are better negotiators and risk averse as documented by [45]. In Malawi and Ethiopia, [40, 46] found that the level of education of value chain players (farmers) has a substantial impact on smallholder farmers’ market channel choice. This suggests that education has a key role in influencing the decision to choose the best option among other alternatives.

#### Unit price

The unit price of the mango in both 2019 and 2020 was measured as a continuous variable [46]. The unit price of a kilogram of mango changes at times with year due to the seasonality of the fruit. When the season fails some mango producers, the price of the fruit changes since it becomes scarce to buy. With this, this study sought to find whether the price offered influences the profit of the value chain actors. The selling price was expressed in Ghana cedis and was determined by the mango market channel (s). For their best benefit, the value chain actors evaluated the price of mango, which influenced their market outlet selection and their profit. This agrees with [47], who found a positive association between avocado price and the amount provided to the market, which was significant at 10%.

#### Volume bought

The quantity or volume of mango purchased could contribute to the decision of who to sell the mango fruit or product to by the mango distributors and processors. Hence, this study expected that the volume of mango fruit or product bought will influence the profit of the producers, distributors, and processors [48].

#### Cooperative or FBO membership

The membership of a cooperative helps the respondents like the producers to obtain a fair price for their produce. The executives of the cooperative meet with the key informants of the marketing outlet, for instance the sales managers of the private processing firms where the price for a kilogram of mango is determined. This makes it possible for the producers not to be cheated by the processing firms with cheaper prices. Membership in an organization was also used as a proxy for access to information. Members are more likely to participate in a sustainable market channel as a result of accessibility of market information than non-members [49]. Membership of Cooperative or FBO as dummy where membership is assigned 1 and no membership is assigned 0.

#### Value addition

It was expected that the value addition of the mango product will positively influence the profit of the mango distributors and processors. It was expected that when these actors add value to the product, the price of the final product will increase and hence influence the specific market outlet ad utility [49].

## Result and discussion

### Governance structure

Table 2 illustrates the perception of the various actors in the mango value chain on the power relational indicators of the governance structure (profit, bargaining power and information concentration). The result revealed that processors were perceived by the other actors (producers and distributors) to have the highest influence in terms of profit (37.67%) earnings and bargaining power (35.38%), while producers were perceived to have the highest influence with respect to information concentration (35.53%). This finding concurs with [24], who found out that in the pig value chain processors are perceived to have the highest influence in terms of profit as compared to distributors and producers.

**Table 2:** Governance in mango value chains.

Governance structure	Producer (%)	Processors (%)	Distributors (%)
Profit	29.87	37.67	32.47
Bargaining power	30.44	35.38	34.18
Information concentration	35.53	34.16	30.30

Source: Survey data

On the contrary, mango producers in the southern part of Ghana are perceived to have the least influence in terms of profit (29.87%) and bargaining power (30.44%). This finding shares a similar thought with [50] where small producers were perceived to have little bargaining power compared with the other actors along the artichoke value chain, which affects their marketing activities and dwindles most importantly their earnings.

The study further revealed that mango distributors in the southern part of Ghana are perceived by other actors to exercise control or influence in terms of profit, bargaining power, and information concentration with a percentage of 32.47%, 34.18% and 30.30%, respectively.

In terms of information concentration, mango producers were perceived as having the highest relative to processors and distributors, whereas the latter (distributors) were perceived to have the least information concentration in the mango value chain in the study area.

### Stakeholder linkages and centrality measures in disseminating market information

**Fig 2:**
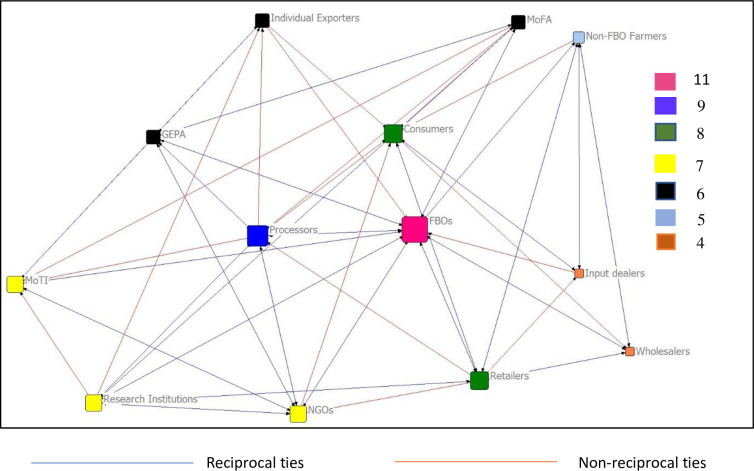
Mapping of actors in the mango value chain.

As represented in Fig 2, Farmer Based Organisations (FBOs), processors, retailers and consumers form the core nodes (stakeholders), while input dealers, wholesalers, and non-FBO farmers are the periphery nodes in receiving market information within the mango value chain. The FBOs are the most central stakeholder with the highest number of ties (11) with other stakeholders in the network. This highlights the FBOs local popularity and activities in dissemination of market information among its members which enhances their bargaining power thus, maximizing their profit. Dissemination of market information and the strong bargaining power serves as one of the incentives for surging membership of the association [51]. It is also important to note that there are as many reciprocal ties than non-reciprocal ties, indicating both forward and backward linkages among actors. Though this is the case, it is important to improve the level of interactions to reduce the number of unidirectional interactions among actors in the network. This will imply actors becoming more proactive in initiating and receiving interactions from other value chain actors.

FBOs which acts as an umbrella over the farmers and has a strong voice in mobilizing collective action and price setting as confirmed in [52] meet at least once a month to provide technical training, share information on market prices, fruit quality, preferred variety in demand and productive logistics (if there is any). They also educate members on how to deal with some existing and possible challenges regarding production and marketing which could retard or promote their activities as observed during the survey and mentioned in [53]. During the interview, support agencies such as the Ministry of Food and Agriculture (MOFA), MOTI, and non-governmental agencies highlighted the importance and usefulness of these meetings since it serves as an avenue for reaching out to the mango producers in their various localities thus, equipping the farmers with the necessary information to boost their productivity and efficiency.

Next to FBOs with a higher number of ties is processors (9), followed by retailers and consumers (8). This reveals the extent of their contribution to receiving market information within the mango value chain. The study further revealed that processors and retailers play a crucial role in ensuring information dissemination due to their contact with a lot of producers and consumers and other stakeholders. However, the stakeholders with the lowest number of ties are input dealers and wholesalers (4), and non-FBO farmers (5). The weak ties position them on the periphery of the network. The small number of wholesalers limits their extent of information dissemination hence their reason for lying on the periphery. The plausible explanation for their less connectedness and interaction with respect to market information dissemination may be due to how they are dispersed and the inability to share market information with other stakeholders.

Further analysis using centrality measures highlighted that FBOs also have the highest in-degree score (91.70) revealing their prominence in the network which is related to how much interactions they receive in the network as compared to other stakeholders (Table 3). In the same light, FBOs recorded the highest outdegree measure (75.00) indicating their influential role in the providing market information to other actors in the network. This is consistent with results for all the other measures of centrality, including betweeness and coreness. FBOs perform a vibrant role in ensuring group cohesion, engagement, and linkages among the stakeholders with regards to individual activities, as noted also by [54, 55]. Their connectedness, roles and activities in market information dissemination scores the highest betweenness and eigen value scores of 42.25 and 39.10 respectively. The highest betweenness score indicates that they serve as the bridging node in the network reflecting their collective action in integrating smallholders into the food and agricultural value chain and the large volume of production by its members [52]. Also, their collective efforts in adhering to internationally recognized standards for good agricultural practices (GLOBALGAP) to produce high quality fruit for both domestic and international market allows their connectedness and contribution to serving as a bridge to other stakeholders within the value chain.

**Table 3:** SNA centrality measures of the value chain actors.

Actor	In-Degree	Out-degree	Betweenness	Coreness	Eigenvalue
FBOs	91.70	75.00	42.25	55.70	39.10
Retailers	41.70	66.70	14.42	17.30	29.40
Research Institutions	41.70	58.30	5.70	34.40	30.00
NGOs	50.00	50.00	4.30	42.10	30.20
Processors	50.00	58.30	5.08	42.50	36.50
Non-FBO Farmers	33.33	41.70	2.78	0.00	17.60
GEPA	41.70	50.00	3.53	31.50	26.00
MOFA	25.00	50.00	1.27	17.00	26.10
Consumers	58.30	25.00	9.33	2.70	27.30
Wholesalers	33.30	25.00	1.28	2.50	15.70
Exporters	33.30	33.30	1.90	5.90	26.10
MoTI	58.30	25.00	1.87	22.10	29.60
Input dealers	25.00	25.00	1.28	0.60	15.70
Network Centralization Index = 28.68%					

Source: Survey data

Consumers, NGOs and processors were the next prominent actors in the network, whilst retailers, research institutions and processors were identified as the next influential actors in the mango value chain network. Retailers being the second top influential actors point out that they are one of the major sources of market information that relates to product quality, price, marketing outlets, variety in demand, and packaging among others which could improve the market efficiency of other actors and maximize their utility. The initiation of interaction in dissemination of market information underlines their connectedness with most of the actors within the mango value chain. This outcome is underscored by the high betweenness score of 14.42 after that of FBO which pinpoints their popularity in initiation of interaction within the mango value chain than they receive from other nodes.

### The impact of COVID-19 on profit of mango value chain actors

The profit earned by the value chain actors in 2019 and 2020 production seasons are presented in Table 4. In 2019 and 2020, the mean profit of mango producers was revealed to be GHS 12215.6 and GHS 8272.4, respectively whilst GHS 5345.4 and GHS 3309.1 were registered for distributors. Processors recorded GHS 14370.6 and GHS 21603.1. The declining profit obtained by producers and distributors of mango could be a result of the COVID-19 disease and its related implications which imperatively disrupted access to inputs, access to current market information, disruption of informal market previously used by the producers and distributors coupled with some exogenous factors like unfavourable climatic conditions for the fruits, post-harvest losses, incidence of pest and diseases, limited labour and transportation among others as indicated by [56]. [57] argued that the result of the COVID-19 shocks on the income level of farm households and consumers forced many to engage in forward-buying of mostly staple food like maize, millet, rice, yam among others to sustain their entire household amidst the COVID-19 pandemic as compared to buying of perishable products like fresh mango, pineapple among others.

**Table 4:** Profit earned by the mango actors between 2019 and 2020.

	Producers (GHS)		Distributors (GHS)		Processors (GHS)	
	2019	2020	2019	2020	2019	2020
Maximum	79934.0	52990.0	31460.0	24958.7	115473.3	139473.3
Average	12215.6	8272.4	5345.4	3309.1	14370.6	21603.1
Minimum	-3871.2	-8102.2	-1312.0	-4607.0	726.0	360.0

Source: Survey data, [1 GHS ≡ USD 11.19, OANDA DATA SERVICES– 04/08/2023]

Processors on the other hand recorded an increase in profit probably due to global surge in fruit and vegetable consumption to boost the immune system against COVID-19 complications as explained by [56, 58]. Even though there were limited number of transport logistics for export like cargo planes, many processors argued that they resorted to the use of any available means of transport like passenger planes to reach their international market. Moreover, the study observed that mango processor’s positive profit could have resulted from the lower price at which they purchased the produce from the producers and, due to the directive by the Government of Ghana and other health related organizations around the world for consumption of fruits and vegetables to boost the immunity against the COVID-19 virus [59].

The revelation by this study with respect to increase in the profit of mango processors during the outbreak of the COVID-19 is consistent with a statement made by an executive member of Blue Skies Company Limited in Ghana who stressed that production volume per week increased significantly as compared to the previous year due to the COVID-19 pandemic which significantly increased their profit earned as a company even though they faced a challenge of high transportation cost resulting from the closure of borders and some restrictions at all ports of entry.

### Impact of COVID-19 on mango actors

Table 5 presents the difference-in-difference estimates revealing the impact of COVID-19 on the profit earned by the mango value chain actors (producers, distributors, and processors). As mentioned above, DID results confirmed that mango producers and distributors had a reduction in profit due to COVID-19 pandemic. The results revealed that there was significant decline by a value of 1.621 and 0.924 for producers and distributors respectively in 2020 (during COVID-19) as compared to 2019 (before COVID-19) probably due to the ubiquitous restrictions especially observed in the Greater Accra region which is the major market destination for many actors in the hinterlands. [13] mentioned that apart from seasonal changes in agriculture production, about 87% of the respondents interviewed for their survey confirmed a decline in profit as a result of the outbreak of COVID-19. In agreement with [60] this paper notes that the decline in profit experienced by producers and distributors due to the COVID-19 response by could be attributed to transport disruption, unregular labour, and limited inputs. Similarly, [61] hinted that the COVID-19 pandemic negatively impacted the profits of rural households due to their vulnerability to economic shocks.

**Table 5:** Difference-in-difference estimates for mango producers, distributors & processors.

	Producers				Distributors				Processors			
Outcome var.	lnProfit	S. Err.	T	P>t	lnProfit	S. Err.	T	P>t	lnProfit	S. Err.	t	P>t
**Before**												
Control	8.807				8.289				8.198			
Treated	8.927				7.532				8.193			
Diff (T-C)	0.12	0.214	0.56	0.575	-0.757	0.31	-2.44	0.016**	-0.005	0.387	-0.01	0.99
**After**												
Control	8.79				7.754				7.813			
Treated	7.289				6.072				8.99			
Diff (T-C)	-1.501	0.221	6.8	0.000***	-1.681	0.362	4.65	0.000***	1.177	0.432	2.73	0.009***
**Diff-in-Diff**												
Diff-in-Diff	-1.621	0.308	5.25	0.000***	-0.924	0.478	1.93	0.056*	1.182	0.55	2.15	0.037**
R-square	0.31				0.35				0.63			

*** p<0.01

** p<0.05

* p<0.1

Source: Survey data

On the other hand, the DID results confirmed a significant positive impact on the profit of processors by a value of 1.182. This could be associated with consumer response to the directives by the government of Ghana and World Health Organization (WHO) on fruit and vegetable consumption to boost immunity against the COVID-19 complications [15, 20]. [60, 62] highlighted an increase in fruit consumption in Sri Lanka by 48.9% in a bit of boosting their immunity and stopping the spread of COVID-19 virus. Also, [63] pointed out that the daily consumption of fruits increased during the outbreak of the COVID-19 as compared to pre-COVID-19. This was among several strategies adopted by people to fight the COVID-19 virus and how it was aggravating other health conditions.

Three variables, which include consumer response, higher output price and packaging were observed to have influenced the outcome of the processors profit at 1%, 5% and 5% significant levels, respectively as seen in Table 6. [64] opined that good packaging is critical to food processing such that it influences profit generated. In their observation, high-quality packaging does not only enhance customers’ perceptions of the reputation of the brand but also the safety of the food and its hygiene. Processors contacted for this study also mentioned how they supplied their products to available supermarkets, malls and retail agent in traffics in response to the surge in quantity demanded by consumers to boost immunity against the COVID-19 virus. The higher demand also led to an increase in the price of the product.

**Table 6:** Impact of covariate on the profits of mango producers, distributors & processors profit.

	Distributors				Processors				Producers			
Variable(s)	Coeff.	Std. Err.	T	P>t	Coeff.	Std. Err.	T	P>t	Coeff.	Std. Err.	t	P>t
*Information access*	0.225*	0.131	1.721	0.088	0.23	0.162	1.421	0.162	0.371**	0.169	2.19	0.029
*Price of inputs*	-0.325*	0.188	-1.73	0.087	0.001	0.0017	-0.586	0.561	-0.561***	0.155	-3.632	0.000
*Consumer response*	0.059	0.143	0.411	0.682	1.040***	0.266	3.907	0.000	0.189**	0.082	2.314	0.021
*Amt*. *of labour*	0.000	0.001	0.102	0.919	-0.161	0.323	-0.498	0.620	-0.005	0.008	-0.635	0.526
*Marital status*	-0.131	0.18	-0.727	0.469								
*Value addition*	0.409*	0.222	1.842	0.068								
*Dist*. *To market*					0.001	0.012	-0.085	0.933				
*Higher price*					0.616**	0.269	2.288	0.027				
*Transport*					-0.065	0.275	-0.237	0.814				
*Packaging*					0.572**	0.282	2.030	0.048				
*Farm size*									0.000	0.009	0.003	0.998
*Herbicide app*.									0.084	0.203	0.413	0.680
Age									-0.002	0.005	-0.389	0.697
Gender									-0.544***	0.146	-3.718	0.000

*** p<0.01

** p<0.05

* p<0.1

Source: Survey data

Other covariates employed in the difference-in-differences (DID) model to assess the impact of COVID-19 on profit generated by the actors revealed a significant negative effect of gender on the profit of producers indicating that the profit earned by male farmers declined by a value of 0.544 compared to what was earned in the previous year. [20] penned that available income or capital to be invested on the farm through pesticide application and other farm management activities were diverted to securing enough food for the household.

Prices of input were found to have a negative impacted on the profit of mango producers and distributors. This revelation might have prevented producers from buying enough farm inputs such as fertilizers, pesticides, and fungicides to enhance productivity. This result is similar to the findings of [59, 65] who explained that many farmers faced difficulty in accessing farm inputs during the outbreak of the COVID-19 which negatively affected their production and profit. Similarly, distributors also lamented the rise in prices of mangoes due to a reduction in production. The consumers’ response revealed an increase in profit of producers of a value 0.189 whilst that of processors also increased by a value of 1.04. Increased in nutritional value by value addition is one marketing factor that attracts more customers which invariably maximizes the returns of the seller [66]. Value addition was adopted as an adaptive strategy during the COVID-19 pandemic and was statistically significant at 10% and reveals a positive impact on the profit of the distributors. This suggests that the distributors who added value to their mango in terms of washing and packaging had the likelihood of increasing their profit by a value of 0.409 as compared to those who did not add value to their fruits.

## Conclusions

The COVID-19 pandemic introduced a challenge to many lives and businesses in the world and for that matter Ghana by stifling agriculture and its related activities, posing significant impact on value chain actors. This requires an empirical study to uncover the extent of the impact posed by the pandemic for better policy considerations. This study therefore assessed the COVID-19 and its impact on the profit of the mango value chain actors in southern Ghana. It also analyzed the governance structure and existing linkages in the dissemination of market information in the mango value chain. The study interviewed 240 respondents in 2020 from Greater Accra, Eastern and Volta regions of Ghana. Net Farm Income, Social Network Analysis and Difference-in-Difference models were used to analyze the data. The governance structure revealed that mango processors have a strong bargaining power and made the most profit followed by distributors within the mango value chain. Also, the mango producers receive more information but are the least profit-making actors within the mango value chain.

The network analysis revealed that Farmer Based Organizations (FBOs), processors, and consumers form the core nodes (stakeholders) while input dealers, wholesalers, and non-FBO farmers were found to be the periphery nodes in receiving market information within the mango value chain. Additionally, analysis using centrality measures highlighted that FBOs have the highest in-degree score revealing their prominence which is related to how much interactions they receive in the network as compared to other stakeholders. In the same light, FBOs recorded the highest outdegree measure indicating their influential role in providing market information to other actors in the network.

Further, mango producers and distributors had a negative profit due to the implementation of restrictions, measures, and policies to contain the COVID-19 spread. However, mango processors were identified to have experienced a positive impact of COVID-19 on their profit. The covariate variables that influenced their profits were consumer response to government directives, prices of input, packaging, gender, higher output price, information access and value addition.

From the ensuing conclusions, our study demonstrated that producers and distributors were vulnerable to the effect of the COVID-19 shock, whilst processors on the other hand, were robust to the shocks. Thus, reformed policies by all stakeholders for emergency preparedness should be targeted especially at those vulnerable actors in the chain. Additionally, the government through the ministry of food and agriculture, FBOs, retailers, and other interested groups should put pragmatic measures that will enhance the dissemination of market information through media and other social network platforms to strengthen the bargaining power of value chain actors whilst improving adaptability in the face of uncertain future for profit maximization.

Though our study uncovered very important findings in relation to the impact of the COVID-19 on mango value chain actors for policy considerations, research into the resilience and post COVID-19 adaptation strategies, and assessment of household food security during the outbreak of the pandemic would be worth investigating.

## Supporting information

S1 Data (XLSX)
